# Tracheobronchial Diverticulosis

**DOI:** 10.5334/jbsr.3771

**Published:** 2024-11-08

**Authors:** Leonor De Almeida Moreira Cameira de Abreu, Jean-Christophe le Brun, Denis Tack

**Affiliations:** 1 Department of Radiology, Epidura La Madeleine, Rue Maria Thomée, 1, 7800 Ath, Belgium

**Keywords:** tracheal wall, diverticulosis, Mounier–Kuhn, tracheomegaly, tomography

## Abstract

*Teaching point:* Tracheal diverticulosis is a rare and benign disorder, characterized by single or multiple tracheal wall outpouchings, either congenital or acquired, usually diagnosed incidentally on chest high‑resolution computed tomography, and in general remaining asymptomatic.

## Case Presentation

A 35‑year‑old female was referred to the emergency department with chronic persistent cough and multiple recurrent respiratory infections. She was a heavy cigarette smoker and further denied fever, chest pain, dyspnea, or weight loss.

**Figure 1:**
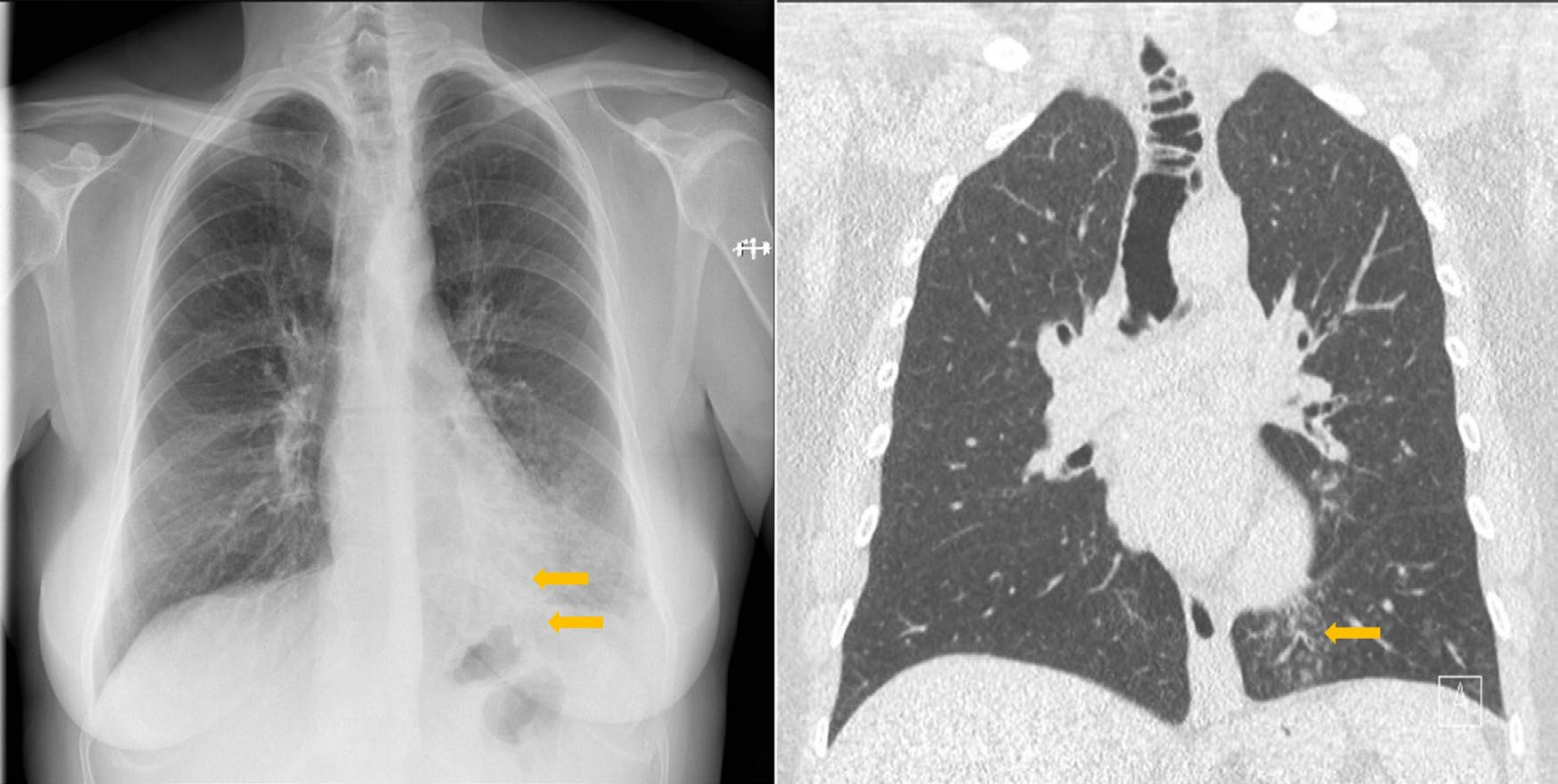
Tracheal diverticulosis and left bronchiolitis; frontal CXR and CT comparison.

Posteroanterior chest X‑ray revealed blurred small pulmonary nodules scattered over the left lower lobe, suggesting lobar bronchiolitis (Figure 1).

After antibiotic therapy for three weeks, a chest computed tomography (CT) examination was performed. Centrilobular nodules with tree‑in‑bud appearance, suggesting inferior lobar bronchiolitis, were still present, but seemed somewhat regressed compared with the initial chest X‑ray. Several tracheal diverticular outpouchings were visible on coronal CT images as well as an increased tracheal size with a maximum transverse diameter of 3.4 cm (Figure 1) consistent with tracheomegaly.

**Figure 2:**
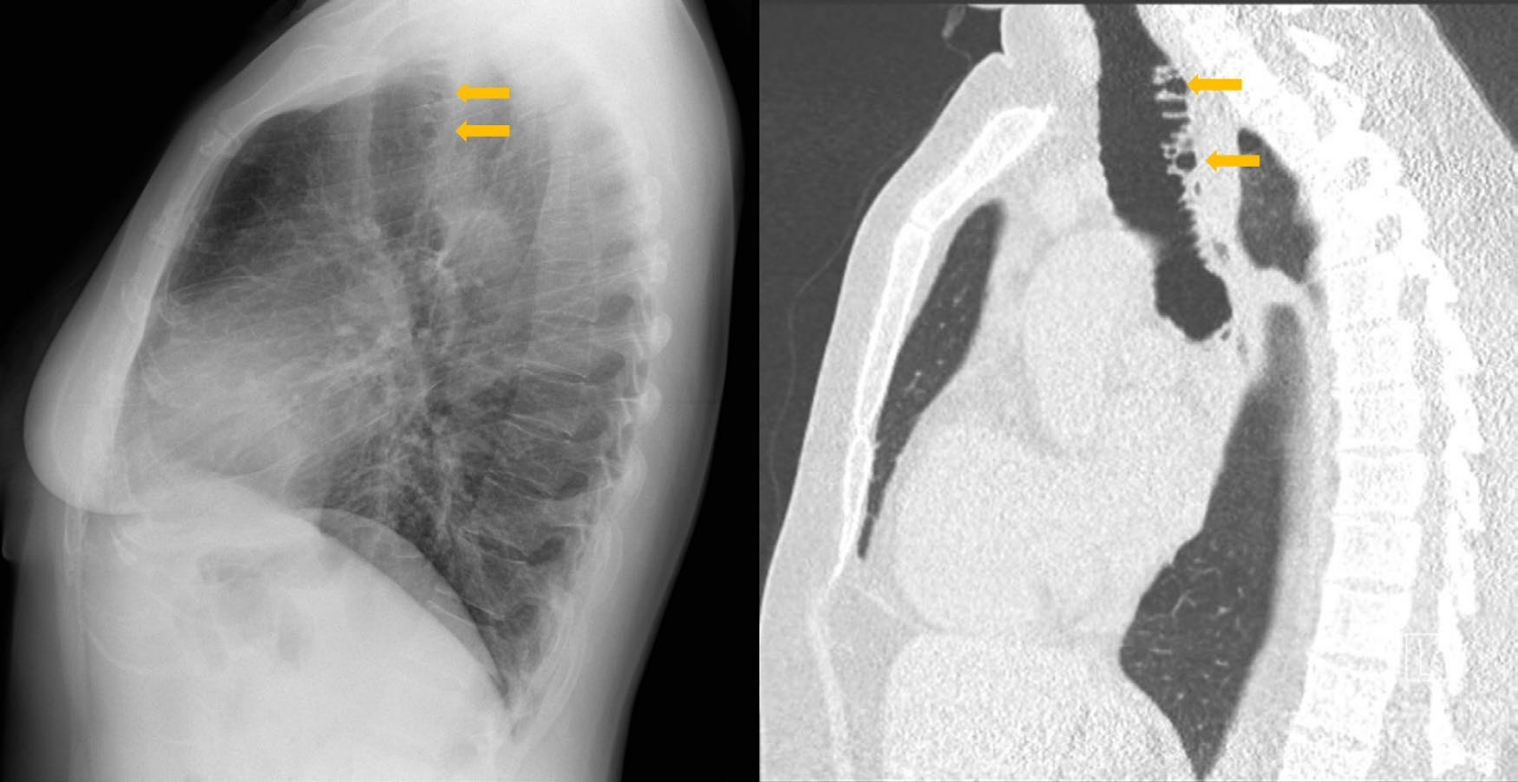
Tracheal diverticulosis: sagital CXR and CT comparison.

On the lateral chest X‑ray (Figure 2), the tracheal diverticulosis was also observable.

**Figure 3:**
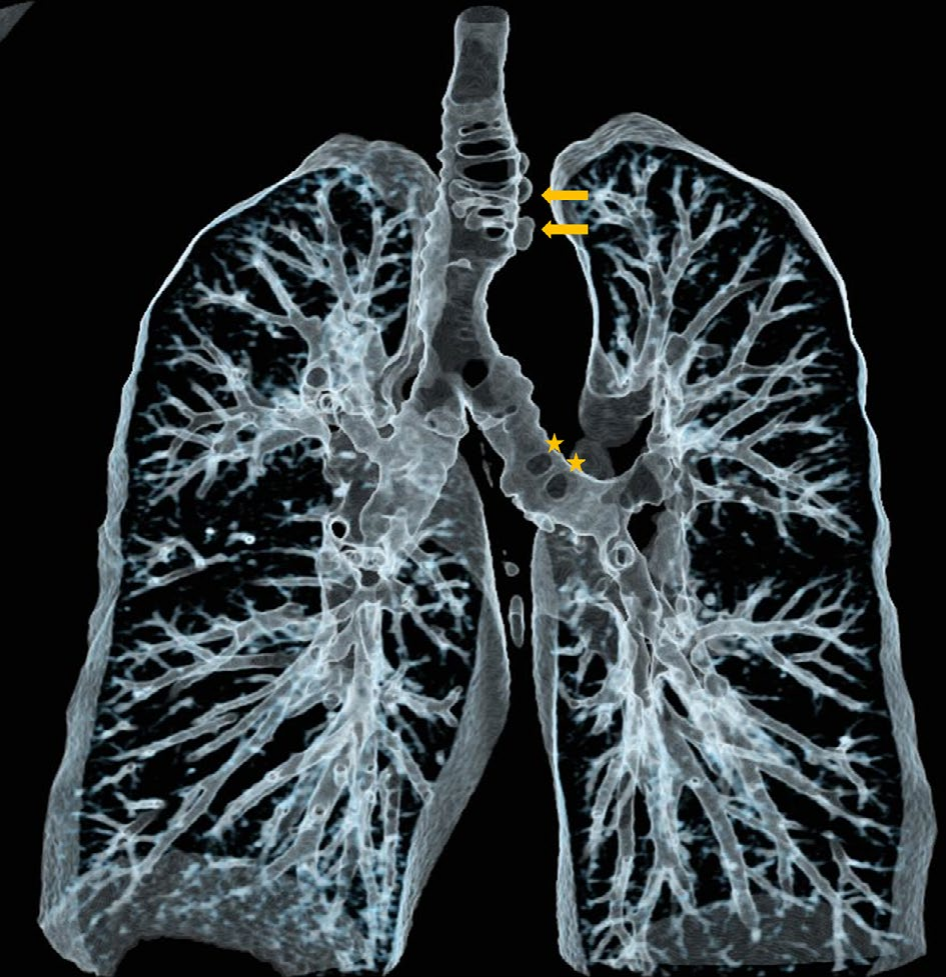
Virtual bronchography of thacheobronchial diverticulosis.

Volume rending technique (VRT) nicely illustrates the distribution of diverticula along the trachea and left main bronchus, as well as more distally in the bronchial tree (Figure 3).

## Discussion

Tracheal diverticulosis is a rare and benign entity defined by single or multiple tracheal wall outpouchings and was first described by Rokitansky in 1838 [1].

Tracheal diverticulosis is generally found incidentally on chest imaging, since the majority of patients are asymptomatic [1]. According to an autopsy series by MacKinnon the overall prevalence is about 1% [1].

Tracheal diverticula can be either congenital or acquired.

Congenital tracheal diverticula are more common in men, are usually found on the right tracheal side, are smaller, and are usually situated approximately 4–5 cm below the vocal cords or just above the carina [1].

In the acquired variant, diverticula may appear at any tracheal level, either as a single diverticulum or multiple diverticula, mostly in the posterolateral area [1]. The acquired subtype with multiple diverticula is a hallmark of tracheobronchomegaly, also referred to as the Mounier–Khun disease [1]. Tracheobronchomegaly is defined by a tracheal diameter exceeding 3 cm, the right main bronchus diameter exceeding 2.4 cm, and the left main bronchus diameter exceeding 2.3 cm.

Rudimentary bronchus, cystic mucus gland duct dilation, tracheocele, and diverticulum associated with tracheobronchomegaly have been described by Katz et al. as the four types of tracheal diverticula [1].

Zenkers diverticulum, laryngocele, pharyngocele, apical lung hernia and bronchogenic cyst are usually listed in the differential diagnosis of tracheal diverticula [1].

Multidetector computed tomography (MDCT) is the preferred imaging modality to demonstrate tracheal diverticulosis and to evaluate the full extent.

Treatment is mainly conservative for asymptomatic patients, with mucolytics, antibiotics, and physiotherapy. Surgical resection may be indicated in symptomatic patients with frequent concomitant infections or for large chronic symptomatic diverticula. Selected cases may be managed with laser endoscopic cauterization [1].

## Informed Consent Statement

Written informed consent was obtained from the patient(s) for publication of this case review, including accompanying images.
